# Measurement Techniques for Highly Dynamic and Weak Space Targets Using Event Cameras

**DOI:** 10.3390/s25144366

**Published:** 2025-07-12

**Authors:** Haonan Liu, Ting Sun, Ye Tian, Siyao Wu, Fei Xing, Haijun Wang, Xi Wang, Zongyu Zhang, Kang Yang, Guoteng Ren

**Affiliations:** 1 Intelligent Microsystems Laboratory, College of Instrument Science and Opto-Electronics Engineering, Beijing Information Science and Technology University, Beijing 100192, China; 2023020357@bistu.edu.cn (H.L.); 2024020371@bistu.edu.cn (Y.T.); 2023030025@bistu.edu.cn (S.W.); zhangzongyu@bistu.edu.cn (Z.Z.); dltkangyang@163.com (K.Y.); 2023020156@bistu.edu.cn (G.R.); 2 Department of Precision Instrument, Tsinghua University, Beijing 100084, China; xingfei@mail.tsinghua.edu.cn; 3 School of Optoelectronic Engineering, Changchun University of Science and Technology, Changchun 130022, China; wanghj@mails.cust.edu.cn; 4 Beijing Institute of Control and Electronic Technology, Beijing 100038, China; 18611689501@163.com

**Keywords:** event cameras, space targets, high-dynamic maneuvers, extraction, star sensor

## Abstract

Star sensors, as the most precise attitude measurement devices currently available, play a crucial role in spacecraft attitude estimation. However, traditional frame-based cameras tend to suffer from target blur and loss under high-dynamic maneuvers, which severely limit the applicability of conventional star sensors in complex space environments. In contrast, event cameras—drawing inspiration from biological vision—can capture brightness changes at ultrahigh speeds and output a series of asynchronous events, thereby demonstrating enormous potential for space detection applications. Based on this, this paper proposes an event data extraction method for weak, high-dynamic space targets to enhance the performance of event cameras in detecting space targets under high-dynamic maneuvers. In the target denoising phase, we fully consider the characteristics of space targets’ motion trajectories and optimize a classical spatiotemporal correlation filter, thereby significantly improving the signal-to-noise ratio for weak targets. During the target extraction stage, we introduce the DBSCAN clustering algorithm to achieve the subpixel-level extraction of target centroids. Moreover, to address issues of target trajectory distortion and data discontinuity in certain ultrahigh-dynamic scenarios, we construct a camera motion model based on real-time motion data from an inertial measurement unit (IMU) and utilize it to effectively compensate for and correct the target’s trajectory. Finally, a ground-based simulation system is established to validate the applicability and superior performance of the proposed method in real-world scenarios.

## 1. Introduction

In spacecraft attitude estimation, star sensors are the most accurate devices available, offering drift-free performance and long operational lifetimes [1,2]. Unlike the coarse estimation methods used during satellite separation, star sensors provide critical support for mission objectives during stable flight through precise measurements, such as accurately aligning with space or Earth target regions. As imaging devices that incorporate advanced image-processing algorithms, star sensors estimate spacecraft attitude by recognizing star distribution patterns—their key function is the accurate extraction of star information from images [3,4,5,6].

As mentioned above, star sensors usually operate during stable flight conditions. However, when used in high-dynamic scenarios—especially during initial orbit insertion, maneuvers, or high-angle attitude adjustments of high-dynamic platforms (such as agile satellites and long-range weapons)—several problems may occur: (1) During the integration period, star points move within the image sensor’s photosensitive area, forming streaks on the sensor’s target plane. This motion blur reduces the image’s signal-to-noise ratio (SNR) and the accuracy of star point centroid localization. (2) Errors in star point centroid localization can lead to redundant or false matching during star map recognition, thereby decreasing both recognition speed and accuracy. (3) Significant positional changes of star points between adjacent frames increase the difficulty in tracking star targets, reducing the success rate and efficiency of star tracking [7,8,9]. Since the 1990s, institutions such as Jena–Optronik (ASTRO–APS) [10] in Germany, AeroAstro (MST) [11] in the United States, and Sodern (SED-16) [12] in France have conducted extensive research on high-dynamic star sensors, achieving a dynamic performance of approximately 10°/s. However, as star sensors are now being applied to high-speed, supersonic, and hypersonic flights, where dynamic rates may reach 15–30°/s, traditional star sensors face severe challenges under these high-dynamic conditions.

Bio-inspired event cameras, as a novel type of sensor, differ from conventional cameras that rely on frame-based imaging modalities. Instead, event cameras can asynchronously capture brightness changes and output event streams containing timestamp, spatial coordinate, and polarity information. Owing to its advantages of microsecond-level temporal resolution, a high-dynamic range (HDR), and low power consumption, this technology has demonstrated significant application potential in space exploration. For instance, in 2017, Cohen et al. successfully detected moving targets in space ranging from low-Earth orbit (LEO) to geosynchronous orbit (GEO) using event cameras in conjunction with large-aperture, long-focal-length ground-based telescopes [13]; in 2019, Chin et al. further explored the application of event cameras in star tracking [14,15]; and in 2021, Roffe et al. validated the reliable operational performance of event cameras in harsh space radiation environments [16].

However, event cameras still face multiple challenges in space target detection applications, with calibration accuracy and high-dynamic-range target extraction emerging as two key technical bottlenecks. In the domain of event camera calibration techniques, Muglikar et al. proposed a general event camera calibration framework based on image reconstruction in 2021, which leverages neural-network-based image reconstruction to break through the limitations of traditional methods that rely on flickering LED patterns [17]. In the same year, Huang et al. developed a dynamic event camera calibration method capable of directly extracting calibration patterns from raw event streams, making it suitable for high-dynamic scenarios [18]. In 2023, Salah et al. developed the E-Calib calibration toolbox, which utilizes the robustness of asymmetric circular grids to handle defocused scenes and introduces an efficient reweighted-least-squares (eRWLS) method for the subpixel-precision extraction of circular features in calibration patterns [19]. In 2024, Salah et al. further advanced the E-Calib toolbox by proposing the eRWLS method, achieving subpixel-precision feature extraction under diverse lighting conditions [20]. Concurrently, Liu et al. introduced the LECalib method, which for the first time, enabled event camera calibration based on line features, particularly suitable for artificial targets, such as spacecrafts with abundant geometric lines [21,22,23]. These advancements in calibration techniques have provided critical support for the application of event cameras in high-precision space measurements.

Event cameras have also demonstrated unique advantages in space target tracking and attitude estimation. In 2023, Jawaid et al. developed the SPADES dataset to address the domain gap issue in satellite attitude estimation, systematically investigating the application of event cameras in space target attitude estimation for the first time [24]. During the Second Satellite Pose Estimation Competition (SPEC2021), organized by Park et al. in 2023, the domain gap between synthetic and real images was highlighted, driving technological progress in this field [25]. In terms of algorithmic innovation, Liu et al. proposed ESVO (Event Stereo Visual Odometry) in 2023, achieving real-time localization and mapping based on stereo event cameras for the first time [26]. In 2024, Wang et al. developed an asynchronous event-stream-processing framework, based on spiking neural networks (SNNs), which suppresses noise through spatiotemporal correlation filtering and achieves target trajectory association using event density features, maintaining stable tracking capabilities even in low signal-to-noise-ratio (SNR) space scenarios [27]. In 2025, Liu et al. proposed a six-degree-of-freedom (6-DOF) pose tracking method for stereo event cameras (stereo-event-based 6-DOF pose tracking). By constructing a target wireframe model and adopting an event-line association strategy, this method enables the continuous pose estimation of non-cooperative spacecrafts. Combined with smoothness-constrained optimization, it resolves the motion blur caused by high-speed movements [22].

In summary, while the algorithmic systems for the event stream processing of event cameras have become relatively comprehensive, there remain notable deficiencies in their dedicated algorithms tailored for space exploration. Specifically, although event cameras have not yet matched the detection accuracy of traditional star trackers for space targets, their performance in high-dynamic scenarios—characterized by microsecond-level temporal resolution and wide-dynamic ranges (WDRs)—significantly surpasses that of conventional cameras, providing a critical complement to high-speed-maneuver target detection. However, in the more challenging context of detecting weak high-dynamic space targets, traditional spatiotemporal correlation filtering methods encounter substantial bottlenecks: They struggle to effectively distinguish target events from background noise. This limitation arises from the inherent characteristics of space targets, which feature weak signals and low signal-to-noise ratios (SNRs), compounded by the energy dispersion induced by high-dynamic maneuvers, which further degrade the target recognition performance of conventional methods. To address this core challenge, this paper proposes an event-driven extraction method for weak high-dynamic targets, aiming to enhance the space target detection capability of event cameras in high-dynamic-maneuver scenarios. The key contributions of this work are summarized as follows:

- For weak space targets’ event stream data, we improve the conventional spatiotemporal correlation filter. Specifically, the event stream is first segmented into pseudo-image frames at fixed time intervals; then, based on the trajectory characteristics of the space targets, a circular local sliding window is employed to determine the spatial neighborhood of the events; finally, noise within the event neighborhoods in the pseudo-image frames is assessed based on the event density—either within a single neighborhood or across multiple neighborhoods—thereby achieving effective denoising of the event stream data for weak space targets (Section 2);
- To extract space targets from the event stream, we introduce a density-based DBSCAN clustering algorithm to achieve subpixel target centroid extraction. For certain ultrahigh-dynamic scenarios—where rapid and nonuniform camera motions lead to target information distortion and data discontinuity—we utilize real-time motion data from an inertial measurement unit (IMU) to construct a camera motion model that infers the camera’s position and orientation at any given moment, thereby enabling the precise compensation and correction of trajectory distortions arising during motion (Section 3);
- We developed a ground-based simulation system to verify the applicability of our proposed method with actual cameras and in real-world scenarios (Section 4).

## 2. Denoising of Weak High-Dynamic Targets

### 2.1. Methodology

**Figure 1:**
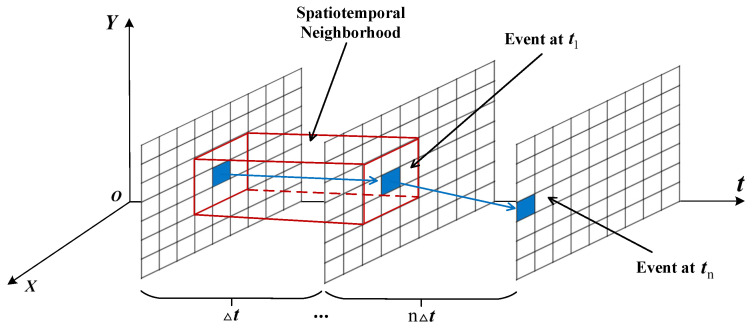
Schematic diagram of the classical spatiotemporal correlation filter principle.

Target events are generated by intensity changes caused by the relative motion between the spacecraft and the target, and the activated pixels are typically adjacent within a certain time period. The distinction between noise and target events lies in the lack of spatiotemporal correlation among noise events within a spatial neighborhood. By exploiting this difference, noise can be filtered out by detecting events produced by neighboring pixels—a process known as the classical spatiotemporal correlation filter (hereafter, the classical filter). As illustrated in Figure 1, the principle is to store the timestamps of events from pixels adjacent to each pixel and then check whether the time difference between the current timestamp and the previous one is less than Δt; if so, the event is retained, otherwise, it is filtered out [28,29].

However, space targets often suffer from weak signals and low signal-to-noise ratios, making it challenging for conventional spatiotemporal correlation filters to differentiate target events from noise in the event stream. To overcome this limitation, we propose an enhanced filtering method that builds on classical filters. In this approach, the event stream is compressed into pseudo-image frames, and a local sliding window is applied to each frame to adjust the spatial neighborhood selection. By leveraging event density information, the filter determines whether an event is noise. This method is termed the Neighborhood-Density-based Spatiotemporal Event Filter (NDSEF). The detailed procedure is as follows:

The NDSEF’s denoising approach begins by sequentially reading the event stream at fixed time intervals to generate pseudo-image frames, denoted as $N_{i}^{t}$, each containing a series of events as follows:

(1) $$N_{i}^{t}=\sum_{(i-1)\Delta t}^{i\Delta t}e\left(x,y,t\right),\;i=1,2,\ldots$$

In the spatial event stream, the motion trajectory of a target signal typically exhibits a roughly cylindrical pattern, and its corresponding local spatial region in a pseudo-image frame tends to be circular or elliptical rather than adopting the rectangular window used in conventional spatiotemporal correlation filters. Therefore, in each pseudo-image frame ($N_{i}^{t}$), we employ a circular local sliding window ($H_{i}^{t}\left(e_{0}\right)$) to iteratively examine every event neighborhood, thereby capturing the spatial distribution characteristics of the target signal more precisely.

(2) $$H_{i}^{t}\left(e_{0}\right)=\{e_{j}\left|\right|e_{j}-e_{0}\left|<r\right\},\;e\in N_{i}^{t}$$

**Figure 2:**
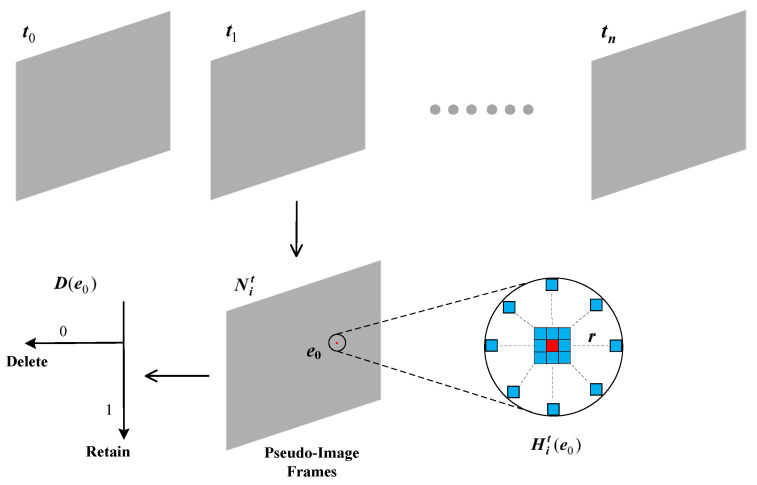
Noise reduction principle of the NDSEF.

As shown in Figure 2, by setting a neighborhood radius (*r*), we calculate the density value ($p(e_{0})$) for the event ($e_{0}$) marked in red in the pseudo-image frame. This density value not only reflects the correlation between events across different timestamps but also reveals the spatial distribution characteristics of events within the local neighborhood. The formula is expressed as follows:

(3) $$p\left(e_{0}\right)=\sum e_{j},\;e_{j}\in H_{i}^{t}\left(e_{0}\right)$$

At each event location, we characterize the sparsity of an event ($e_{0}$) by the accumulated event density ($p(e_{0})$) over a fixed time interval (Δt). By applying an event density threshold (dTh) to filter out noise, the filtering outcome is represented by a binary function (*D*), where 1 denotes a target event, and 0 denotes a noise event. The mathematical expression is as follows:

(4) $$D\left(e_{0}\right)=\left\{\begin{array}{cc} 1, & \mathrm{if}\;d\left(e_{0}\right)\geq d_{Th}\\ 0, & \mathrm{if}\;d\left(e_{0}\right)<d_{Th} \end{array}\right.,\;e_{0}\in N_{i}^{t}$$

### 2.2. Optimization

While the aforementioned method achieves satisfactory denoising in low-dynamic space target scenarios, it encounters challenges when an aircraft is in a high-dynamic maneuvering state. Under such conditions, the spatial distribution of target events becomes elongated within an extremely short time. In these cases, the NDSEF method—which relies solely on a fixed-scale neighborhood radius (*r*) for density calculations—tends to preserve noise due to the larger neighborhood, thereby significantly reducing its denoising performance.

To address the deterioration in the denoising performance under high-dynamic maneuvering conditions, we introduce multiple neighborhood radii ($\left\{r_{1},r_{2},\ldots ,r_{K}\right\}$) (ordered from the smallest to the largest) to the same pseudo-image frame, conducting a neighborhood search and density estimation at each scale. Subsequently, the results across different scales are fused to determine whether a particular event should be retained or discarded. The primary steps and formulae are described as follows:

1. Define a set of radii $R=\{r_{1},r_{2},\ldots ,r_{K}\}$, where $0<r_{1}<r_{2}<\ldots <r_{K}$. For each event ($e_{0}$) in the pseudo-image frame ($N_{i}^{t}$), we define its neighborhood set ($H_{i}^{k}\left(e_{0}\right)$) at different radii as follows:
  $$H_{i}^{t}\left(e_{0}\right)\;=\;\left\{\;e_{j}\;|\;e_{j}\in N_{i},\;\;∥\left(x_{j},y_{j}\right)-\left(x_{0},y_{0}\right)∥<r_{k}\right\},\;k=1,2,\ldots ,K;$$
2. At each radius ($r_{k}$), the local neighborhood density ($p_{k}\left(e_{0}\right)$) is defined as follows:
  $$p_{k}\left(e_{0}\right)\;=\;\sum_{\;e_{j}\;\in \;H_{i}^{t}\left(e_{0}\right)}1\;=\;\left|\;H_{i}^{k}\left(e_{0}\right)\right|$$
  The above expression indicates the number of events within the *k*th-scale neighborhood that are sufficiently close to $e_{0}$;
3. To integrate the density information across multiple scales, we introduce a multiscale fusion function ($M(e_{0})$) to describe the overall density of event $e_{0}$ within neighborhoods defined by different radii. The expression below indicates that only those events that maintain a certain density across all the scales are recognized as target events, thereby effectively eliminating false detections caused by local noise aggregation.
  $$M\left(e_{0}\right)\;=\;\min_{\;1\leq k\leq K}\;\left\{\;p_{k}\left(e_{0}\right)\right\};$$
4. In a manner similar to that in the single-scale filtering approach, we need to define a final decision threshold ($M_{\mathrm{th}}$) to determine whether an event ($e_{0}$) is retained (denoted as 1) or discarded (denoted as 0).

Multiscale neighborhood fusion, indeed, enhances the filtering accuracy for weak targets under high-dynamic conditions, but it also incurs additional computational overhead. To ensure the real-time performance, we adopt the following optimization strategies:

- Hierarchical screening: First, a rapid preliminary screening is performed using a small radius ($r_{1}$) to eliminate the vast majority of the sparse noise; only the events retained from this initial screening are then subjected to a more refined density evaluation using larger radii ($r_{2},r_{3},\ldots$);
- Parallel/neighborhood accumulation: When computing the density across various scales, an octree spatial data structure is employed, enabling a single query to return the aggregation count for multiple radii, thereby reducing redundant scanning.

## 3. Extraction of Weak High-Dynamic Targets

Compared to traditional frame cameras, such as star sensors, event cameras exhibit significantly higher temporal resolutions, with a response speed of up to 1 microsecond/event [30]. This high temporal resolution effectively mitigates target position extraction bias caused by motion blur in high-dynamic scenarios. In this study, we convert the event stream to pseudo-image frames at fixed time intervals and apply a density-based DBSCAN (Density-Based Spatial Clustering of Applications with Noise) clustering algorithm to precisely extract subpixel centroids of moving space targets [31,32].

The DBSCAN clustering algorithm relies on two key parameters: the neighborhood radius and the minimum number of samples per event cluster. In pseudo-image frames, the extraction of target centroids depends on the clustering performance of the DBSCAN algorithm, and its mathematical expression is given as follows:

(5) $$\left(x_{c},y_{c}\right)=\frac{1}{\left|N\right|}\sum_{i=1}^{\left|N\right|}\left(x_{i},y_{i}\right)$$

In the equation, the centroid’s coordinates ($\left(x_{c},y_{c}\right)$) are computed as the weighted average of the spatial coordinates of all the events within each event cluster. Each centroid represents the target’s position in the current frame, and by analyzing the variation in the centroid’s positions across consecutive frames, the target’s motion trajectory can be effectively tracked.

**Figure 3:**
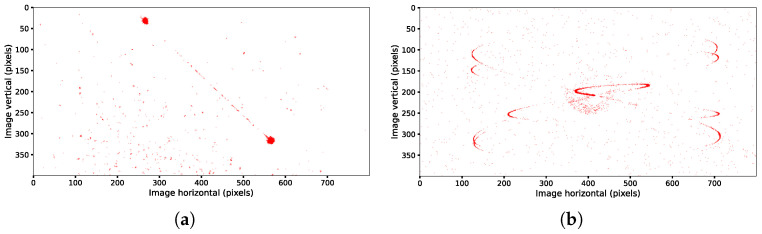
Schematic of the target’s trajectory after optimized-time single-channel filtering. (**a**) Linear motion with an angular velocity of 20°/s; (**b**) sinusoidal motion with an amplitude of 10° and a frequency of 1 Hz.

However, under certain ultrahigh-dynamic conditions, the target trajectories captured by the event camera are often distorted due to the camera’s extremely rapid and nonuniform motion. The acquired data exhibit significant discontinuities with large gaps, which compromise the accuracy of the centroid extraction, as shown in Figure 3.

To compensate for the trajectory distortion caused by the camera’s ultrahigh-speed motion and to improve the accuracy of the target centroid extraction, we incorporate camera acceleration and angular velocity data provided by an inertial measurement unit (IMU) to infer and correct the camera’s trajectory. Currently, the DVXplorer Lite event camera from ETH Zurich is equipped with an integrated IMU system [33]. The real-time motion information supplied by the IMU facilitates the construction of an accurate camera motion model, which allows us to determine the camera’s position and orientation at any given moment and achieve precise compensation for the trajectory distortions incurred during motion.

The camera’s motion consists of translational and rotational components. The accelerometer and angular velocity data from the IMU can be used to describe these motions [34,35]. The acceleration ($a\left(t\right)={\left[a_{x}\left(t\right),a_{y}\left(t\right),a_{z}\left(t\right)\right]}^{T}$) measured by the accelerometer allows us to calculate the camera’s velocity and position. Assuming the camera’s initial velocity at time $t_{0}$ is $v_{0}$ and its initial position is $P_{0}$, by integrating the acceleration, we can calculate the camera’s velocity (v(t)) and position (P(t)) at any time (*t*) as follows:

(6) $$v\left(t\right)=v_{0}+\int_{0}^{t}a\left(t^{\prime }\right)dt^{\prime }$$

(7) $$P\left(t\right)=P_{0}+\int_{0}^{t}v\left(t^{\prime }\right)dt^{\prime }=P_{0}+\int_{0}^{t}\left(v_{0}+\int_{0}^{t^{\prime }}a\left(t^{\prime \prime }\right)dt^{\prime \prime }\right)dt^{\prime }$$

This method calculates the camera’s positional change by doubly integrating the acceleration. The angular velocity ($\omega \left(t\right)={\left[\omega_{x}\left(t\right),\omega_{y}\left(t\right),\omega_{z}\left(t\right)\right]}^{T}$), measured by the gyroscope, describes the camera’s rotational speed around the three axes. By integrating the angular velocity, the camera’s orientation (R(t)) can be obtained as follows:

(8) $$R\left(t\right)=R_{0}\cdot \exp\left(\int \left[\omega \left(t^{\prime }\right)\right]\times dt^{\prime }\right)$$

where $R_{0}$ is the initial orientation of the camera, and $\omega (t^{\prime })$ is the angular velocity measured at time $t^{\prime }$. Integrating the angular velocity gives the camera’s rotational change in three-dimensional space.

Due to differences in the sampling frequency between the acceleration and angular velocity data provided by the IMU and the event points captured by the event camera, the event camera records information from rapidly changing dynamic scenes, while the IMU data are used to infer the camera’s motion state at each moment. In practical applications, we employ an interpolation method to adjust the IMU data to match the event camera’s sampling times. After time synchronization, the IMU’s acceleration and angular velocity data can be accurately aligned with each event point captured by the event camera, thereby providing camera motion parameters for every event.

Using the motion information provided by the IMU, we can correct the event points. Let $e_{k}\left(t\right)=\left(t,x_{k},p_{k}\right)$ represent an event captured by the camera at time *t*, where $x_{k}$ is the event’s position in the image, and $p_{k}$ is the event’s polarity (enhancement or suppression). Using the IMU’s motion data, and assuming the camera’s motion (position and orientation) state at the event capture’s time is P(t) and R(t), we can use the following formula to correct the event point’s spatial coordinates:

(9) $$x_{k}^{\prime }=T\left(x_{k},t;P\left(t\right),R\left(t\right)\right)$$

where $x_{k}^{\prime }$ is the corrected position of the event point, *T* is the compensation model, and P(t) and R(t) represent the camera’s position and orientation at time *t*, respectively.

**Figure 4:**
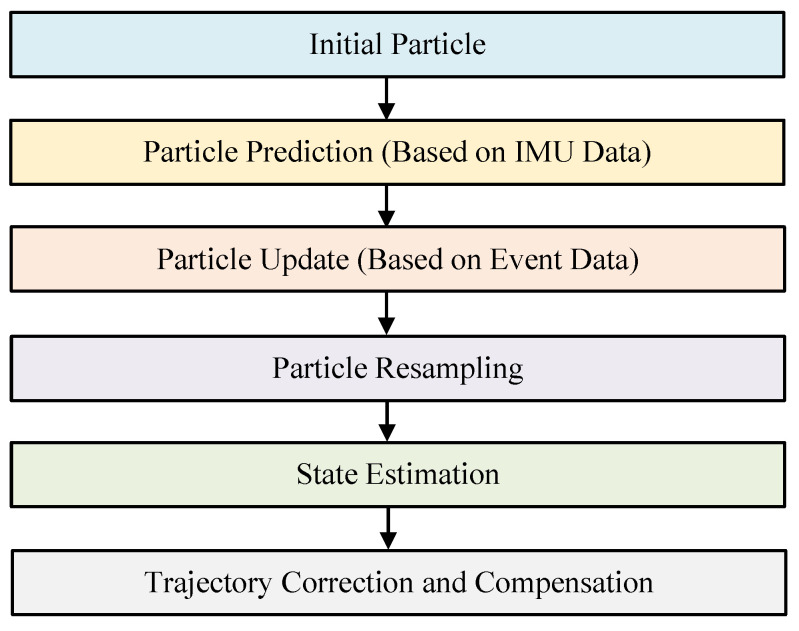
Flowchart of the particle-filtering algorithm.

According to the above, we have developed a method that estimates the camera’s motion using IMU data to compensate for the high-dynamic trajectories of star points. However, the IMU sensor itself introduces high-frequency random noise under dynamic conditions and may accumulate biases and drifts over prolonged use, due to factors such as zero offset and temperature variations. To further improve the accuracy of the target trajectory’s compensation, this paper introduces a particle-filtering algorithm to correct compensation errors caused by noise or sensor offsets. Particle filtering is a state estimation algorithm, based on Monte Carlo methods, which estimates the true state of the system by incorporating multiple hypothesized states (particles) and assigning a weight to each, thereby overcoming the limitations of traditional Kalman filtering in handling nonlinearities and non-Gaussian noise. The computational flowchart for the particle-filtering algorithm is shown in Figure 4.

1. Initializing the Particle Set: The first step in particle filtering is initializing a set of particles, each representing a possible state of the camera. Each particle represents motion parameters, such as position, velocity, and orientation. These particles can be initialized using the IMU’s initial data or randomly based on a prior motion model. The state of each particle is represented as $x_{k}={\left[P_{k},v_{k},R_{k}\right]}^{T}$, where $P_{k}$ is the position, $v_{k}$ is the velocity, and $R_{k}$ is the orientation;
2. Particle Prediction (Based on IMU Data): At each time step (*k*), the particles predict the current state based on the previous state and the IMU’s acceleration ($a(t_{k})$) and angular velocity ($\omega (t_{k})$). This process is executed through the motion model.
  (10) $$\left\{\begin{array}{c} v_{k}=v_{k-1}+a\left(t_{k}\right)\Delta t\\ P_{k}=P_{k-1}+v_{k}\Delta t\\ R_{k}=R_{k-1}\cdot \exp\left(\left[\omega \left(t_{k}\right)\right]\times \Delta t\right); \end{array}\right.$$
3. Particle Update (Based on Event Data and Observations): Particle filtering’s core is updating particles’ weights using the event camera’s observational data. Each particle’s weight is proportional to how closely its predicted state matches the actual observational data. For each particle (*i*), the match with the event camera data is calculated to determine the particle weight ($w_{k}^{\left(i\right)}$). Particles with higher match values receive higher weights.
  (11) $$w_{k}^{\left(i\right)}=\frac{p(z_{k}|x_{k}^{\left(i\right)})}{\sum_{j=1}^{N}p\left(z_{k}|x_{k}^{\left(j\right)}\right)}$$
  where $z_{k}$ is the observational data from the event camera, and $p(z_{k}|x_{k}^{\left(i\right)})$ is the match between the particle and event data;
4. Particle Resampling: Particle filtering resamples the particles based on their weights, creating a new particle set. This eliminates low-weight particles and focuses on those that better match the actual observational data, improving accuracy and robustness;
5. State Estimation: The optimal estimate at the current time is obtained by calculating the weighted average of the particles’ states (${\hat{x}}_{k}$). Particle filtering computes the camera’s final motion state by averaging the states of all the particles, weighted by their respective weights, as follows:
  (12) $${\hat{x}}_{k}=\sum_{i=1}^{N}w_{k}^{\left(i\right)}x_{k}^{\left(i\right)};$$
6. Trajectory Correction and Compensation: Using the optimal estimated state, the trajectory of star points captured by the event camera can be corrected. Particle filtering compensates for lost or distorted event points, corrects deviations in the camera’s trajectory, and restores the original target event’s trajectory.

## 4. Experiments and Results

### 4.1. Experimental Setup

Experimental targets primarily consist of high-dynamic objects in space. Most of these targets are at great distances and appear as point-like objects on the imaging plane, while a subset are extended targets. Notably, the point-like targets occupy from only a few to a dozen pixels in the captured images, closely resembling the appearance of distant stars. Therefore, the design of the light source’s brightness in the experiment can be referenced to stellar magnitude standards.

**Figure 5:**
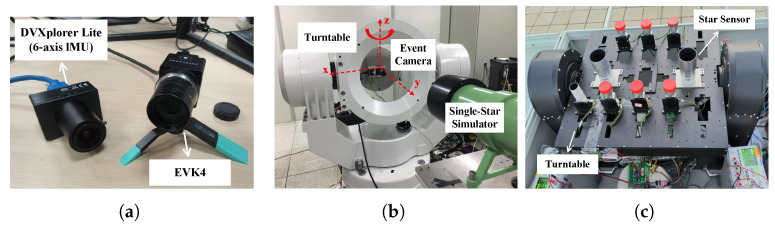
Experimental environment and equipment. (**a**) The event camera used in the experiment (DVXplorer Lite: Basel, Switzerland’s iniVation, EVK4: Atsugi, Japan’s Sony and PROPHESEE); (**b**) High-dynamic-measurement experimental setup (Turntable: State Key Laboratory of Precision Measurement Technology and Instruments, Tsinghua University, Beijing, China); (**c**) Real night stargazing experimental setup (Turntable and Star Sensors: State Key Laboratory of Precision Measurement Technology and Instruments, Tsinghua University).

Since starlight can be regarded as parallel light, a single-star simulator was constructed using a combination of an LED light source, a mask, and a collimator. Specifically, the single-star simulator generates parallel light, with a brightness similar to that of a star, through the LED light source, uses the mask to adjust the spot size, and then transmits the beam to the event camera’s field of view through the collimator. To ensure that the parallel light emitted by the single-star simulator adequately covers the field of view of the event camera, the event camera is mounted on a high-precision turntable that is aligned parallel to the single-star simulator, as shown in Figure 5b. In addition, for the brightness calibration of the LED light source in the single-star simulator, the experiment referenced nighttime observational data from a star sensor. Figure 5c shows the experimental setup for nighttime star observation, which provided the basis for the light source calibration in this experiment.

In this study, the aforementioned experimental apparatus was employed to conduct multiple experiments within an angular velocity range of 4∼20°/s. Detailed analyses and comparisons of the noise reduction performances and centroid extraction accuracies of the proposed method were carried out, thereby investigating the capability of the event camera to detect space targets under high-dynamic conditions.

The proposed event-based method for extracting weak high-dynamic targets involves multiple parameters, which optimization critically determines algorithmic feasibility; this section provides implementation guidance for optimizing the key parameters.

- Temporal Interval (Δt): Event cameras typically operate at microsecond temporal resolutions. Conventional spatiotemporal denoising methods applied at these resolutions risk event loss during high-dynamic imaging. To mitigate this, our algorithm integrates events into pseudo-image frames, using Δt intervals calibrated against typical star tracker exposure durations (e.g., 5 ms, 10 ms, and 20 ms). This ensures sufficient space target events are captured per frame while maintaining high-temporal-resolution advantages;
- Neighborhood Parameters (*r*, *R*) and Density Threshold ($d_{Th}$): These values were empirically determined through the statistical analysis of experimental data across 4–20°/s angular velocities, with scenario-adaptive adjustments: Stronger motion dynamics (reducing the target energy) necessitate larger radii (*r*) and lower density thresholds ($d_{Th}$), while elevated stray light conditions require smaller radii (*r*) and higher thresholds ($d_{Th}$). Spatial distribution analysis under varying angular velocities yielded the following statistically optimal parameters: neighborhood radius set *R* = 3, 5, 7, 9, 11, *…* pixels and density threshold $d_{Th}$ = 25–100 pixel densities (equivalent to 5 × 5–10 × 10 pixel areas) based on high-dynamic star-imaging characteristics—target events are identified when neighborhood event counts exceed these thresholds.

### 4.2. Experimental Results and Analysis

**Figure 6:**
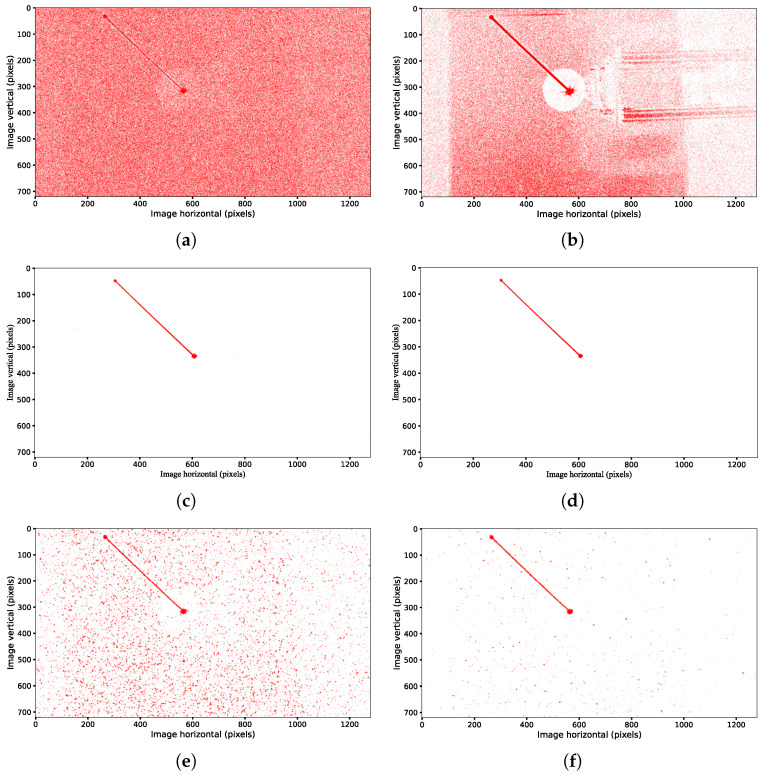
Denoise processing and optical-flow compensation diagram. (**a**) Original image at 10°/s. (**b**) Classical filter at 10°/s. (**c**) NDSEF filter at 10°/s. (**d**) MR-NDSEF filter at 10°/s. (**e**) NDSEF filter at 20°/s. (**f**) MR-NDSEF filter at 20°/s.

Figure 6 presents a comparison of the denoising performances among the classical filter, the NDSEF filter, and the multi-neighborhood-radius-based NDSEF filter (referred to as MR-NDSEF) in various angular velocity scenarios, with a constant light source brightness (equivalent to that of a magnitude-three star). Notably, the results in Figure 6e,f are based on IMU data for target trajectory compensation, and their performances can be compared with the compensation results shown in Figure 3.

From Figure 6a–d, it can be observed that when the angular velocity is relatively low in high-dynamic maneuvers, the classical filter, although capable of preserving target signal events, still leaves a significant amount of noise after filtering, thereby increasing the difficulty in the subsequent target extraction. In contrast, both the NDSEF filter and the MR-NDSEF filter markedly improve the denoising performance—the target signals appear much clearer, and the use of a circular local sliding window better conforms to the characteristics of the target signals’ trajectory in the spatial data. Moreover, the results in Figure 6e,f indicate that when the camera operates at higher angular velocities, the energy of the target signal is further diminished, and the MR-NDSEF filter exhibits an even superior denoising performance.

Evaluating the denoising performance requires not only qualitative visual assessments but also scientifically rigorous and comprehensive quantitative metrics. To facilitate the quantitative evaluation of the noise filters, this paper employs the signal-to-noise ratio (SNR) for quantitative analysis. The SNR reflects the energy ratio between the target signal and the noise before and after filtering and is defined by the following equations:

(13) $$SNR^{o}=10\times {\mathrm{lg}}_{10}\frac{E_{signal}^{o}}{E_{noise}^{o}}$$

(14) $$SNR^{f}=10\times {\mathrm{lg}}_{10}\frac{E_{signal}^{f}}{E_{noise}^{f}}$$

In the equations, $E^{f}$ represents the number of events after denoising, $E^{o}$ represents the number of events before denoising, signal is the original event stream, noise is the event stream with added noise, $SNR^{o}$ denotes the signal-to-noise ratio before denoising, and $SNR^{f}$ denotes the signal-to-noise ratio after denoising.

**Figure 7:**
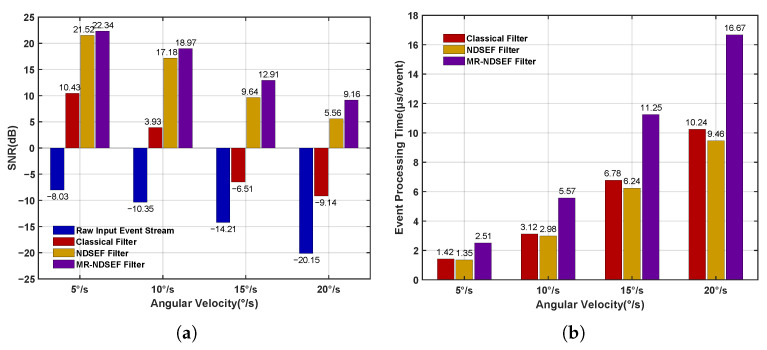
Evaluation of the noise reduction effect. (**a**) Signal-to-noise ratio (SNR). (**b**) Event-processing time.

Figure 7 quantitatively evaluates the filtering qualities of the classical filter, the NDSEF filter, and the MR-NDSEF filter, using two metrics: the signal-to-noise ratio (SNR) and event-processing time. As shown in the figure, after denoising with both the NDSEF and MR-NDSEF filters, the signal-to-noise ratio of the space target’s event stream is significantly improved. Notably, at relatively high angular velocities, the MR-NDSEF filter exhibits a particularly outstanding filtering performance. Furthermore, the event-processing time of the NDSEF filter is comparable to that of the classical filter, while the MR-NDSEF filter requires a relatively longer processing time, which is generally maintained at approximately 10 µs, thereby satisfying the real-time requirements of space exploration.

**Figure 8:**
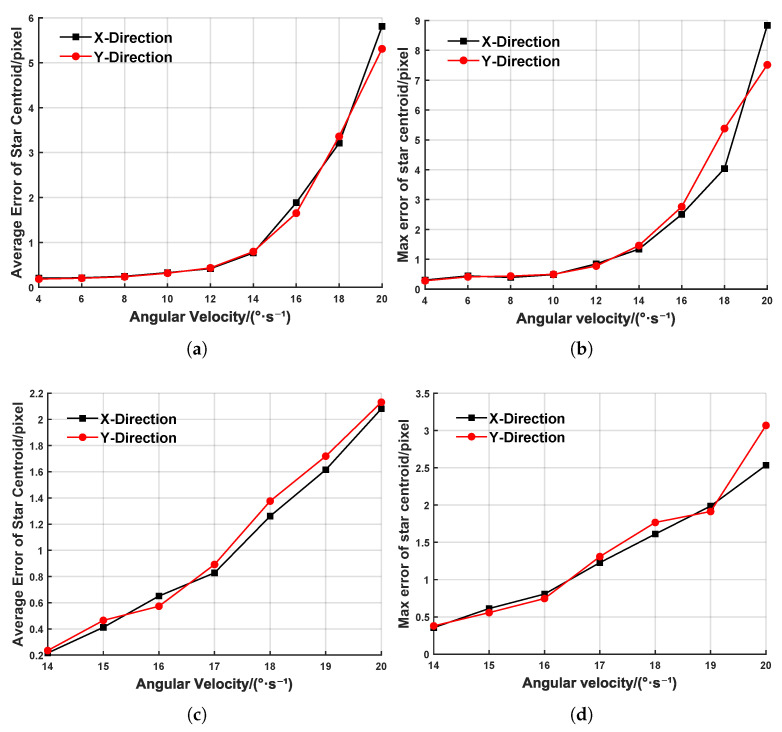
Error of centroid extraction at different angular velocities. (**a**) Average error of centroid extraction. (**b**) Maximum error of centroid extraction. (**c**) Average error of centroid extraction (after IMU compensation). (**d**) Maximum error of centroid extraction (after IMU compensation).

To validate the effectiveness of the DBSCAN algorithm and the IMU-based event compensation method for the extraction of target centroids under high-dynamic conditions, we selected 500 ms event streams with a stable angular velocity and a stable acceleration for each angular velocity, extracted the target centroids, and computed the average and maximum errors between the extracted target centroids and the theoretical ground truth, as shown in Figure 8. In Figure 8a,b, when the dynamic angular velocity is in the range 4∼12°/s, the average target centroid’s error is less than 0.5 pixels, and the maximum error remains at the subpixel level. However, when the dynamic angular velocity increases to 12∼20°/s, the extremely rapid and nonuniform camera motion leads to missing target events and gaps between events, causing both the average and maximum centroid errors to surge drastically. At 20°/s, the average error exceeds five pixels, which, theoretically, renders it as unsuitable for space target exploration. Figure 8c,d presents the target centroids’ extraction errors after IMU-based event compensation, clearly showing a significant improvement in the extraction accuracy. At 20°/s, the average error is reduced by 65% and the maximum error by 71%, thereby demonstrating the effectiveness of the IMU-based compensation method in mitigating missing events under extremely high-dynamic conditions.

**Figure 9:**
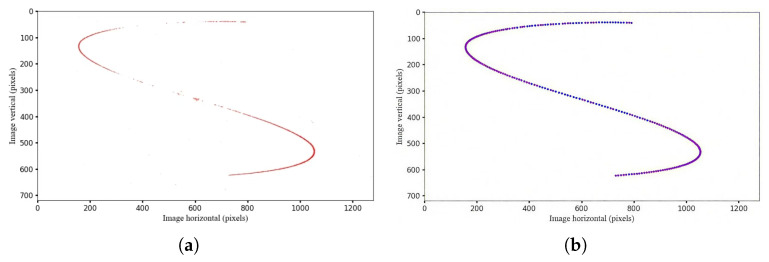
Event camera sinusoidal motion event acquisition trajectory (Noise Reduction). (**a**) Before event compensation. (**b**) After event compensation.

All the experiments described above were conducted under conditions of a constant angular velocity. To further validate the reliability of our method for target centroid extraction, we analyzed an event stream collected while the camera underwent sinusoidal motion at varying frequencies and amplitudes (with non-constant angular velocities and accelerations). Figure 9a shows the event stream acquired under sinusoidal motion with an amplitude of 10° and a frequency of 1 Hz, while Figure 9b presents the target centroid results extracted from the event stream after applying event compensation based on IMU data.

**Figure 10:**
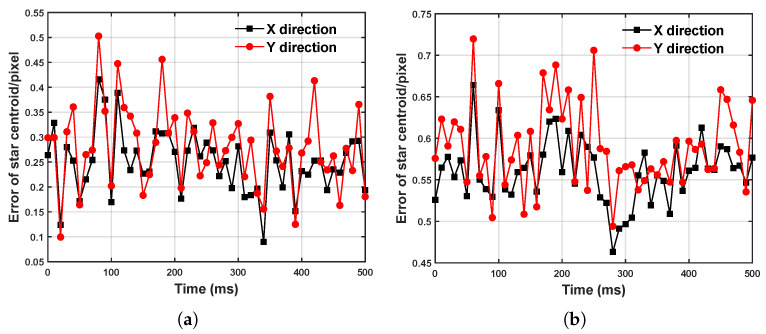
Error of centroid extraction under sinusoidal motion. (**a**) The first 500 ms event data stream. (**b**) The second 500 ms event data stream.

In this event stream, we randomly selected two segments of 500 ms event data and calculated the average error between the extracted centroid and the theoretical ground truth for each segment. As shown in Figure 10a,b, under nonlinear and nonuniform motion conditions, the average error in the target centroid extraction remains at the subpixel level. This indicates that even under complex motion conditions, the event compensation method based on IMU data can effectively enhance the accuracy of the target extraction.

## 5. Verification

**Figure 11:**
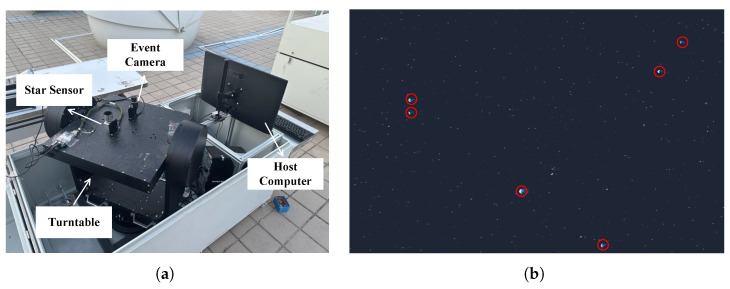
Nighttime star observation experiment. (**a**) Experimental equipment. (**b**) Experimental results (Red circles mark the identifiable navigation stars).

In the validation case, we conducted an outdoor stellar observation experiment, as illustrated in Figure 11a. In this experiment, a star sensor and an event camera were co-mounted on a rotary table. By leveraging the well-established target recognition algorithm of the stellar sensor, celestial objects were precisely identified, enabling the calibration of the equivalent stellar magnitudes of the targets captured by the event camera. Figure 11b presents the experimental results of this outdoor stellar observation, demonstrating the feasibility of using event cameras for target detection in space.

**Figure 12:**
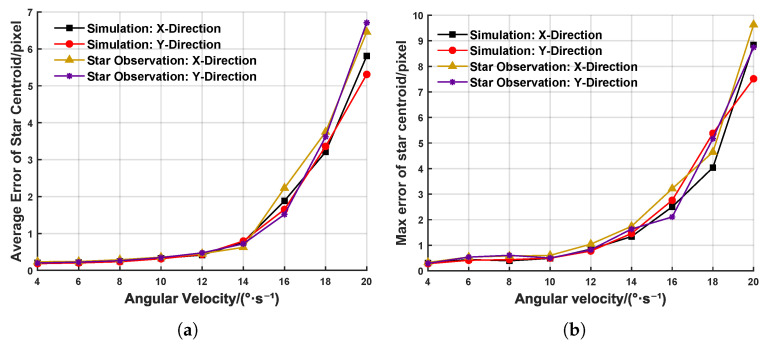
Error of centroid extraction at different angular velocities in ground simulation and stargazing experiments. (**a**) Average error of centroid extraction. (**b**) Maximum error of centroid extraction.

By leveraging the existing equipment and resources, we conducted multiple stellar observation experiments and compared the experimental results with those from ground-based simulation experiments (targeting magnitude-three stars). The error distributions are shown in Figure 12. These results validate the effectiveness and reliability of the proposed event-based extraction method for weak high-dynamic targets.

## 6. Conclusions

In this paper, we proposed an event data extraction method tailored for weak, high-dynamic targets. Unlike existing event target extraction algorithms, our approach fully accounts for the trajectory characteristics of moving targets during the event-stream-denoising stage by employing the multiscale neighborhood density for noise determination, thereby significantly enhancing the signal-to-noise ratios of high-dynamic space targets. In the target extraction phase, we not only integrate the DBSCAN clustering algorithm but also address issues of trajectory distortion and data discontinuity in ultrahigh-dynamic scenarios by constructing a camera motion model using real-time motion data from an inertial measurement unit (IMU) to compensate for and correct the target’s trajectory. The experimental results validate the robustness and reliability of our algorithm in both denoising and target extraction. Future work will focus on the following directions: (1) extending the algorithms to accommodate multitarget high-dynamic application scenarios tailored for practical space exploration missions, (2) designing large-aperture lenses integrated with event cameras and continuing to conduct ground-based high-dynamic stellar observation experiments to investigate the ultimate detection capabilities of event cameras, and (3) developing a high-dynamic motion dataset for space targets, integrating machine-learning techniques to perform in-depth studies on sparse event stream data, and achieving adaptive parameter selection for the algorithms.

## Data Availability

The synthetic data supporting the results of this study are available from the corresponding author upon reasonable request.

## References

[1] Xing F., You Z., Sun T., Wei M. (2017). Principle and Implementation of APS CMOS Star Tracker.

[2] Liu H.N., Sun T., Wu S.Y., Yang K., Yang B.S., Ren G.T., Song J.H., Li G.W. (2025). Development and validation of full-field simulation imaging technology for star sensors under hypersonic aero-optical effects. Opt. Express.

[3] Liebe C. (2002). Accuracy performance of star trackers—A tutorial. IEEE Trans. Aerosp. Electron. Syst..

[4] Yang B., Fan Z., Yu H. (2020). Aero-optical effects simulation technique for starlight transmission in boundary layer under high-speed conditions. Chin. J. Aeronaut..

[5] Liu H., Sun T., Yang K., Xing F., Wang X., Wu S., Peng Y., Tian Y. (2025). Aero-Thermal Radiation Effects Simulation Technique for Star Sensor Imaging Under Hypersonic Conditions. IEEE Sens. J..

[6] Li T., Zhang C., Kong L., Wang J., Pang Y., Li H., Wu J., Yang Y., Tian L. (2024). Centroiding Error Compensation of Star Sensor Images for Hypersonic Navigation Based on Angular Distance. IEEE Trans. Instrum. Meas..

[7] Wang J. (2019). Research on Key Technologies of High Dynamic Star Sensor. Ph.D. Thesis.

[8] Wan X., Wang G., Wei X., Li J., Zhang G. (2021). ODCC: A dynamic star spots extraction method for star sensors. IEEE Trans. Instrum. Meas..

[9] Zeng S., Zhao R., Ma Y., Zhu Z., Zhu Z. (2022). An Event-based Method for Extracting Star Points from High Dynamic Star Sensors. Acta Photonica Sin..

[10] Schmidt U. ASTRO APS-the next generation Hi-Rel star tracker based on active pixel sensor technology. Proceedings of the AIAA Guidance, Navigation, and Control Conference and Exhibit.

[11] Cassidy L.W. (1993). Miniature star tracker. Proceedings of the Space Guidance, Control, and Tracking.

[12] Foisneau T., Piriou V., Perrimon N., Jacob P., Blarre L., Vilaire D. (2017). SED16 autonomous star tracker night sky testing. Proceedings of the International Conference on Space Optics—ICSO 2000.

[13] Cohen G., Afshar S., Morreale B., Bessell T., Wabnitz A., Rutten M., van Schaik A. (2019). Event-based sensing for space situational awareness. J. Astronaut. Sci..

[14] Chin T.J., Bagchi S., Eriksson A., Van Schaik A. Star tracking using an event camera. Proceedings of the IEEE/CVF Conference on Computer Vision and Pattern Recognition Workshops.

[15] Bagchi S., Chin T.J. Event-based star tracking via multiresolution progressive Hough transforms. Proceedings of the IEEE/CVF Winter Conference on Applications of Computer Vision.

[16] Roffe S., Akolkar H., George A.D., Linares-Barranco B., Benosman R.B. (2021). Neutron-induced, single-event effects on neuromorphic event-based vision sensor: A first step and tools to space applications. IEEE Access.

[17] Muglikar M., Gehrig M., Gehrig D., Scaramuzza D. How to calibrate your event camera. Proceedings of the IEEE/CVF Conference on Computer Vision and Pattern Recognition.

[18] Chen S., Li X., Yuan L., Liu Z. (2025). eKalibr: Dynamic Intrinsic Calibration for Event Cameras From First Principles of Events. arXiv.

[19] Salah M., Ayyad A., Humais M., Gehrig D., Abusafieh A., Seneviratne L., Scaramuzza D., Zweiri Y. (2024). E-calib: A fast, robust and accurate calibration toolbox for event cameras. IEEE Trans. Image Process..

[20] Peng X., Wang Y., Gao L., Kneip L. (2020). Globally-optimal event camera motion estimation. Proceedings of the Computer Vision–ECCV 2020: 16th European Conference.

[21] Liu Z., Liang S., Guan B., Tan D., Shang Y., Yu Q. (2025). Collimator-assisted high-precision calibration method for event cameras. Opt. Lett..

[22] Liu Z., Guan B., Shang Y., Bian Y., Sun P., Yu Q. (2025). Stereo Event-based, 6-DOF Pose Tracking for Uncooperative Spacecraft. IEEE Trans. Geosci. Remote Sens..

[23] Cieslewski T., Choudhary S., Scaramuzza D. Data-Efficient Decentralized Visual SLAM. Proceedings of the 2018 IEEE International Conference on Robotics and Automation (ICRA).

[24] Elms E., Jawaid M., Latif Y., Chin T. (2022). SEENIC: Dataset for Spacecraft PosE Estimation with NeuromorphIC Vision. https://zenodo.org/records/7214231.

[25] Park T.H., Märtens M., Jawaid M., Wang Z., Chen B., Chin T.J., Izzo D., D’Amico S. (2023). Satellite Pose Estimation Competition 2021: Results and Analyses. Acta Astronaut..

[26] Liu Z., Shi D., Li R., Zhang Y., Yang S. (2023). T-ESVO: Improved event-based stereo visual odometry via adaptive time-surface and truncated signed distance function. Adv. Intell. Syst..

[27] Wang R., Wang L., He Y., Li L. (2024). A space point object tracking method based on asynchronous event stream. Aerosp. Control Appl..

[28] Liu H., Brandli C., Li C., Liu S.C., Delbruck T. (2015). Design of a spatiotemporal correlation filter for event-based sensors. Proceedings of the 2015 IEEE International Symposium on Circuits and Systems (ISCAS).

[29] Gouda M., Abreu S., Bienstman P. (2024). Surrogate gradient learning in spiking networks trained on event-based cytometry dataset. Opt. Express.

[30] Raviv D., Barsi C., Naik N., Feigin M., Raskar R. (2014). Pose estimation using time-resolved inversion of diffuse light. Opt. Express.

[31] Çelik M., Dadaşer-Çelik F., Dokuz A.Ş. (2011). Anomaly detection in temperature data using DBSCAN algorithm. Proceedings of the 2011 International Symposium on Innovations in Intelligent Systems and Applications.

[32] Deng D. (2020). Research on anomaly detection method based on DBSCAN clustering algorithm. Proceedings of the 2020 5th International Conference on Information Science, Computer Technology and Transportation (ISCTT).

[33] Stewart T., Drouin M.A., Picard M., Djupkep Dizeu F.B., Orth A., Gagné G. A virtual fence for drones: Efficiently detecting propeller blades with a dvxplorer event camera. Proceedings of the International Conference on Neuromorphic Systems 2022.

[34] Ahmad N., Ghazilla R.A.R., Khairi N.M., Kasi V. (2013). Reviews on various inertial measurement unit (IMU) sensor applications. Int. J. Signal Process. Syst..

[35] Yan H., Shan Q., Furukawa Y. RIDI: Robust IMU double integration. Proceedings of the European Conference on Computer Vision (ECCV).

